# Lessons learnt from developing and applying research priorities during the COVID-19 pandemic: reflections from the Global Research Collaboration for Infectious Disease Preparedness (GloPID-R)

**DOI:** 10.1136/bmjgh-2024-015278

**Published:** 2024-07-30

**Authors:** Emilia Sitsofe Antonio, Moses Alobo, Alice Norton, Charles S Wiysonge

**Affiliations:** 1 GloPID-R Research and Policy Team, Policy & Practice Research Group, Pandemic Sciences Institute, University of Oxford, Oxford, UK; 2 Science For Africa Foundation, Nairobi, Kenya; 3 South African Medical Research Council, Tygerberg, South Africa; 4 World Health Organization Regional Office for Africa, Brazzaville, Congo

**Keywords:** COVID-19, Health policy, Health systems evaluation

**Summary box**

- In response to the COVID-19 pandemic, multiple activities to identify priority areas for research were undertaken.
- Existing guidelines for development of research priority agendas are limited in discussion of priority setting in the context of epidemic/pandemic response.
- We present key recommendation on best practice for developing and applying research priority agendas during outbreaks.
- These considerations represent key learnings from the COVID-19 pandemic, which can strengthen preparedness for responding to future epidemics and pandemics.

## Background

 The global response to the COVID-19 pandemic has seen many funders supporting research across multiple disciplines to gain key insights into various aspects of the pandemic.^1^ Given the novelty and impact of COVID-19, it has been essential for research to be directed to the most urgent research questions for controlling the pandemic.

The Global Research Collaboration for Infectious Disease Preparedness (GloPID-R), a global network of funders of preparedness and response research for outbreaks of emerging and re-emerging pathogens, has been undertaking activities to strengthen collaboration and coordination during the COVID-19 pandemic. In February 2020, GloPID-R coconvened the Global Research and Innovation Forum with WHO resulting in a Global Research Roadmap for COVID-19, which outlined immediate and mid-term to long-term research priority areas for focusing the global research effort.^2^ This roadmap has been instrumental in setting the agenda for research for global COVID-19 prevention and control.^3^

Multiple further research priority setting activities employing varying methodologies were undertaken during the pandemic. These include institutional, national and regionally led activities such as undertaken by the African Academy of Sciences (AAS), Africa Centres for Disease Control and Prevention (Africa CDC), WHO African Regional Office and African Union Development Agency-New Partnership for Africa’s Development to identify the COVID-19 research priorities of relevance to Africa,^4^ and related activities to outline COVID-19 research priorities for LMICs^4^,^6^ informing both local AAS funding calls and international funding calls (eg, the UK Global Effort on COVID-19 health research call).

GloPID-R’s Research in Low-and-Middle-Income Countries (LMICs) Working Group was formed in the early stages of the pandemic to support collaboration of the GloPID-R membership for a coordinated approach to COVID-19 research in LMICs.^7^ Since February 2022, the Working Group has been evaluating the research prioritisation activities undertaken regionally and globally in response to COVID-19. As the pandemic wanes, it is important to highlight the key recommendations for developing and applying research priorities emerging from this work to inform improved preparedness for disease outbreaks in the future. The LMIC Working Group recently published a report on ‘lessons learnt from developing and applying research priorities during the COVID-19 pandemic’^8^ and here we highlight the key recommendations for consideration by the global health community.

## Emerging recommendations from working group consultations

- **Rapid prioritisation is essential for rapid research initiation:** the need to act promptly in the response to outbreaks is especially urgent when there are limited infection countermeasures to save lives. Prompt prioritisation is important for guiding the efficient allocation of resources for research. One recommended approach to avoid delays in identifying and convening suitable experts for the assessment of research gaps is for experts to be assembled and prepositioned in the interpandemic period in readiness for a new outbreak and engaged rapidly at the onset. The value of activities for identifying priority areas for research in advance of epidemics, such as WHO R&D Blueprint mechanism, was also highlighted.
- **Comprehensive research prioritisation methods promote trust in research agenda:** efforts to expedite priority setting activities in response to outbreaks must not be at the expense of the quality of methods employed. The application of systematic approaches aligning to existing health research priority setting standards^9^ promotes the credibility of research agenda developed and the likelihood of necessary stakeholder engagement (funders, researchers, affected populations and other policy stakeholders).
- **Inclusivity of voices is essential for capturing diverse perspectives:** for research priorities to be truly representative of their identified remit of global, regional, national or local research needs the relevant ‘voices’ must be heard in priority setting activities. It is important to consider the representation of regional and local voices in the development of global research agenda as well as marginalised and vulnerable groups.
- **Flexible funding policies are key for the application of research priorities to funding decisions:** predesign of adaptable, nimble policies in pandemic response mode to allow for pivoting of existing research activities were identified as key to a rapid response. Furthermore, supplementary funding, repurposing grants and coordinated funding among multiple funders are required for implementation of priority agenda developed.
- **Monitoring and evaluation to enable living research roadmaps:** it is crucial for assessment processes for progress against research agenda to be promoted particularly in a fast-moving pandemic where research needs rapidly evolve. This allows for the detection of persistent research gaps and facilitates the funding of novel research questions, which are relevant as new evidence becomes available. Prospective planning for monitoring and evaluation can be facilitated by building in feedback loops in the planning for research priority setting activities.
- **Transparency and communication must underpin research priority setting processes:** the cross-cutting themes of transparency and communication emerged as key recommendations for fostering trust in research funding systems for disease outbreaks. Here, it was recommended that stakeholders involved in research prioritisation processes: articulate a clear purpose for the research agendas, including the target audience; be transparent in criteria for shortlisting priority areas; and, be open about the mode of application of these to funding decisions.

## Discussion and recommendations

These recommendations complement existing broad standards for health research priority setting, adding new context relevant to infectious disease outbreaks, with a focus on regional and global priority setting. The recommendations are applicable in multiple settings and many are of particular importance to LMICs where the efficient use of limited resources is of the essence.

Africa’s regional approach was the only emerging example of regional research prioritisation at the time of the consultations. COVID-19 research priorities for the WHO South-East Asia region have since been published in September 2022.^10^ Further work to identify standards for priority setting tailored to epidemic and pandemic contexts is still required to extrapolate beyond COVID-19. The PSI Policy and Practice Research Group is undertaking a scoping review on prioritisation activities for high consequence pathogens to map existing practice in this field beyond COVID-19.^11^

## Conclusion

From consultations on lessons learnt from research prioritisation during the COVID-19 pandemic, the GloPID-R Research in LMICs Working Group has identified key recommendations for developing and applying research priorities crucial for improving disease outbreak preparedness and response in the future. We invite funders, researchers and other stakeholders to engage with the recommendations presented here.

## Data Availability

Data are available upon reasonable request.
